# Genome-wide association mapping in winter barley for grain yield and culm cell wall polymer content using the high-throughput CoMPP technique

**DOI:** 10.1371/journal.pone.0173313

**Published:** 2017-03-16

**Authors:** Andrea Bellucci, Alessandro Tondelli, Jonatan U. Fangel, Anna Maria Torp, Xin Xu, William G. T. Willats, Andrew Flavell, Luigi Cattivelli, Søren K. Rasmussen

**Affiliations:** 1 Department of Plant and Environmental Sciences, Faculty of Sciences, University of Copenhagen, Frederiksberg, Denmark; 2 Consiglio per la Ricerca e la Sperimentazione in Agricoltura e l’Analisi dell’Economia Agraria, Centro di Ricerca per la Genomica Vegetale, Fiorenzuola d’Arda, Italy; 3 School of Life Science, University of Dundee, Dundee, United Kingdom; GERMANY

## Abstract

A collection of 112 winter barley varieties (*Hordeum vulgare* L.) was grown in the field for two years (2008/09 and 2009/10) in northern Italy and grain and straw yields recorded. In the first year of the trial, a severe attack of barley yellow mosaic virus (BaYMV) strongly influenced final performances with an average reduction of ~ 50% for grain and straw harvested in comparison to the second year. The genetic determination (GD) for grain yield was 0.49 and 0.70, for the two years respectively, and for straw yield GD was low in 2009 (0.09) and higher in 2010 (0.29). Cell wall polymers in culms were quantified by means of the monoclonal antibodies LM6, LM11, JIM13 and BS-400-3 and the carbohydrate-binding module CBM3a using the high-throughput CoMPP technique. Of these, LM6, which detects arabinan components, showed a relatively high GD in both years and a significantly negative correlation with grain yield (GYLD). Overall, heritability (*H*^*2*^) was calculated for GYLD, LM6 and JIM and resulted to be 0.42, 0.32 and 0.20, respectively. A total of 4,976 SNPs from the 9K iSelect array were used in the study for the analysis of population structure, linkage disequilibrium (LD) and genome-wide association study (GWAS). Marker-trait associations (MTA) were analyzed for grain yield and cell wall determination by LM6 and JIM13 as these were the traits showing significant correlations between the years. A single QTL for GYLD containing three MTAs was found on chromosome 3H located close to the Hv-eIF4E gene, which is known to regulate resistance to BaYMV. Subsequently the QTL was shown to be tightly linked to rym4, a locus for resistance to the virus. GWAs on arabinans quantified by LM6 resulted in the identification of major QTLs closely located on 3H and hypotheses regarding putative candidate genes were formulated through the study of gene expression levels based on bioinformatics tools.

## Introduction

Barley (*Hordeum vulgare* L.) is a major grain crop in Europe and the fifth most-produced crop worldwide after maize, rice, wheat and soybean (FAOSTAT, 2016). Barley is used for food, feed, in the malting industry and as a speciality crop due to health claims. Recent advancements in genome sequencing techniques are now also available for barley using high-throughput platforms for genotyping germplasm collections at a relatively low cost [1, 2]. This allowed the utilization of a genome-wide association study (GWAS) to dissect the genetics mechanisms behind agriculturally relevant traits and identify markers that can be used in breeding programs to improve cultivar performance. Single genes such as *INT-C* regulating spike morphology in barley have been identified using GWAS [3] but this technique has also proved to be extremely efficient for studying complex quantitative traits. Frost tolerance has recently been analysed using GWAS to detect several associated markers in a collection of 184 barley genotypes [4]. Similarly, 140 marker-trait associations (MTAs) were detected for yield and malting quality traits using historical records of 174 spring and winter barley varieties [5].

Plant cell wall composition and the properties of its constituents have attracted interest in the scientific community over the past decade for several reasons. The increasing number of genomic resources available coupled with highly efficient techniques of quantification and observation of plant cell wall fine structures have boosted knowledge in this area [6, 7]. Plant cell walls provide support to reproductive organs, structures for nutrients relocation, and resistance to pathogen and abiotic stresses. Its role is thus fundamental in crop production [8]. Furthermore, improving bioenergy production using ligno-cellulosic material requires a better understanding of how plant cell walls are synthesized and how it is possible to obtain crop biomass characterized by less recalcitrance to enzymatic saccharification [9]. Plant cell walls are made of polysaccharides, mainly cellulose, hemicellulose and pectins, and phenolic compounds, such as in lignin. They are divided into primary and secondary cell walls and can vary in composition according to species, developmental stages and the tissue considered. The primary cell wall is found in a highly hydrated state, allowing flexibility and cell adhesion in growing cells. In grasses, where all the relevant cereal crops are found, the cell wall is constituted mainly of cellulose (20–30% dry weight), hemicellulose (heteroxylans and mixed-linkage glucans, 30–70% dry weight) and pectins (5%). In secondary cell walls, found in mature, non-elongating cells, lignin (20% dry weight) replaces the pectic fraction, providing rigidity and resistance against stresses, thanks also to the reduced amount of water present due to its characteristic hydrophobicity. Furthermore, the majority of hemicellulose is constituted of heteroxylans [10]. Minor amounts of arabinogalactan proteins can also be detected. For comparison, dicots do not synthesize mixed-linkage glucans and the hemicellulosic fraction of the primary cell wall is mainly constituted of xyloglucans and pectins. Additionally, dicots have a lower amount of lignin in the secondary wall (7–10%) [11]. A specific characteristic of grass cell walls is the presence of ferulic and p-coumaric acid responsible for crosslinking hemicellulose-lignin and lignin units respectively. From a genetic standpoint the complexity of plant cell walls implies the involvement of a great number of genes co-expressed during plant development and it is estimated that ~10% of the entire plant genome is devoted to cell wall biosynthesis and modification [9]. Most knowledge about genes involved in polysaccharide composition is based on reverse and forward genetic studies in *Arabidopsis thaliana*. Among the most relevant are the cellulose synthase family A (*CesA*) genes coding for the macro-complex responsible for cellulose deposition [12] and the cellulose synthase like (*Csl*) superfamily including several genes responsible for the synthesis of mixed-linkage glucans, xyloglucans and mannans [13]. Nevertheless, many genes involved in carbohydrate metabolic pathways, e.g. glycosyl transferase (GT) and glycosyl hydrolase (GH) families are still poorly characterized (see http://www.cazy.org for details). Furthermore, there are additional classes of transcription factors such as the MYB and NAC gene families acting as master switches for cell wall deposition and modification in response to biotic and abiotic stresses [14] delineating a complex landscape of gene interactions, as recently shown in Arabidopsis [15].

In light of this, a genome-wide approach can represent a powerful tool to assist the identification of candidate genes involved in plant cell wall biosynthesis. However, very few examples of GWAS on traits related to lignocellulosic biomass composition and properties are available. A combined approach using QTL mapping and GWAS was recently employed to identify candidate genes related to 2^nd^ generation biofuel production in maize [16], distinguishing several GT and transcription factors, and the release of monomeric sugars in wheat [17]. Energy crops such as *Miscanthus sinensis* [18], reed canary grass [19] and sweet sorghum [20] are also currently being investigated for traits related to biomass composition and biofuel production and studies are reporting several MTAs that further improve the genetic material available. In barley, candidate genes involved in culm cellulose content have been identified as belonging to the *CesA* gene family [21]. Two likely explanations for such low numbers of GWAS in traits related to plant cell walls are that: *i)* the variance due to genetic effects detectable for such traits is usually low due to the plasticity that plants have in order to respond to the wide range of inputs from the environment; *ii)* phenotyping plant cell walls is usually a complex task that require time consuming, labour-intensive and skill demanding techniques and it is not always possible to screen the hundreds of samples required to obtain a sufficient number of data points to perform GWAS.

In this context, the comprehensive microarray polymer profiling (CoMPP) technique appeared to be one of the most suitable high-throughput methods for characterizing a collection of samples for cell wall composition and subsequent GWAS [22]. Thanks to the great number of monoclonal antibodies (mABs) and carbohydrate-binding domains (CBMs) available that recognize specific polysaccharide epitopes, this technique has been employed successfully in studies of plant evolution, plant physiology and bioethanol production [23, 24]. Here we present a GWA mapping study of a collection of 112 European 2-row and 6-row winter barley varieties tested in field trials for two years and characterized for grain and straw yield and culm cell wall composition using the CoMPP technique. The collection was genotyped using a high-density SNP array and analysed for population structure and linkage disequilibrium (LD). Finally, potential candidate genes were selected based on QTL identified through GWAS results, using bioinformatics resources and previous knowledge of the traits studied.

## Materials and methods

### Plant material, field trials and phenotyping

The germplasm collection consisted of 112 winter barley (*Hordeum vulgare* L.) accessions representing cultivated barley from 11 countries in Europe released by breeding companies and public institutions over the last 60 years, with a few even older than that. Of these, 58 were two-row and 56 were six-row type accessions. Three accessions lacked a year of release and two were missing a country of origin (see supplementary material).

Field trials were sown in 2008 and 2009 and harvested in 2009 and 2010 (they will be referred to below only by their year of harvest) at CREA (Center for Agricultural Research and Economics of Italy)—Genomics Research Centre of Fiorenzuola d’Arda (44°55'44.3"N, 9°54'09.7"E) in Italy. No specific permissions were required to conduct the trials since only barley varieties were involved and no endangered or protected species were included in the study. It should be noted that the field trials were physically separated from one another. The dimension of each plot was 2 m^2^ using a completely randomized block design for each year and standard agronomical practices for the location. Due to adverse weather conditions in autumn 2008, sowing was delayed until 11 November, while the 2009 sowing occurred on 30 October. Prior to grain harvesting barley plants were manually harvested from 1 linear meter of the in middle section of the central row of each plot. Spikes were then removed, straw was air-dried and the total straw weight was recorded for each plot subsample (SYLD). Afterwards, the field plots were harvested using a combined automatic harvester and the grain yield (GYLD) was recorded. A sample of the straw collected (~ 40 g) was subsequently manually separated into leaves and culms and while the former was not included in further analysis the latter was kept for cell wall polysaccharide characterization. Culms from each plot were ground to < 1 mm particles on a cyclone mill and stored in sealed plastic containers. For the analysis, a custom designed robotic platform (Labman Automation Ltd., United Kingdom) was utilized for fine grinding, feeding and weighing samples into 96-well plate format racked collection microtubes (product code: 19560, Qiagen, Germany). The platform can handle 288 different samples in a single run, moving them to different stations using robotic arms. Samples are tracked using barcode labels applied to each sample container and recorded automatically throughout the entire process. The main steps of the process are: *i) grinding*, each sample is loaded in a screw-cap sealed 20 ml plastic vial with five metal beads and moved from the starting position to the grinding station where a robotic arm shakes the vial at ~5000 rpm until a fine powder is obtained; *ii) declogging and piercing*, the vial is repeatedly inverted to mix the powder then, while upside-down, the lid is pierced and moved to the feeding station; *iii) feeding*, a 96-well plate is moved on the balance with customized support (Sartorius WZ614-CW, Sartorius AG, Germany). The vial is placed by a robotic arm on the defined well and shaken allowing the powder to fall into the well. The weight is constantly recorded until the target of 10 ± 0.3 mg (average water content 8.5 ± 0.7%) is reached. Once the feeding process is completed, the vial and plate are carried to their initial position and a new sample is processed. The quantification of cell wall components with antibodies was carried out using the Comprehensive Microarray Polymer Profiling (CoMPP) technique as described in Moller et al. [22]. Briefly, starting from fine-ground plant material in the 96-well microtiter plate, the cell wall polymers were obtained by first extracting pectins with 50 mM diamino-cyclo-hexan-tetra-acetic acid (CDTA), pH 7.5, and then extracting polysaccharides (hemicelluloses and cellulose) with 4M NaOH. A third extraction step using cadoxen (31% v/v 1,2-diaminoethane with 0.78 CdO) included in the original protocol was omitted based on Alonso-Simon et al. [24]. Supernatants containing solubilized cell wall polymers were diluted 2–10- and 100- fold in PBS (140 mM NaCl, 2.7 mM KCL, 10 mM Na_2_HPO_4_, 1.7 mM KH_2_PO_4_, pH 7.5) and printed in duplicates on a nitrocellulose membrane on the same day as the extraction using a microarray robot (Arrayjet Sprint Inkjet Microarrayer, http://www.arrayjet.co.uk/). In total each sample was represented by 6 spots (replicates). Given the physical dimension of the membrane, a maximum of 114 samples plus 6 standards for calibration between membranes were included in each extraction. The standards were constituted by two different samples from the same set of winter barley culms analyzed, extracted and printed in triplicates each time and used as a reference for subsequent data normalization. After all the arrays were printed, probing was performed using monoclonal antibodies (mABs) and a carbohydrate-binding module (CBM) specific for binding to α (1→ 5)-arabinan (mAB LM6), β (1→4)-xylan-arabinoxylan (mAB LM11), β(1→3)(1→4)-glucan (mAB BS-400-3), arabinogalactan protein-glycan (mAB JIM13) and crystalline cellulose (CMB3a). They were chosen based on a preliminary study in which a subset of the samples was probed with a more extended list of mABs or CBMs, selecting those showing higher variation and reproducibility (data not shown). Probing with primary antibodies was performed as described in Alonso-Simon et al. [24] and detection performed using conjugated antibodies. Probed and developed arrays were scanned using a high resolution flat-bed scanner (Canon 9950F) at 2400 dpi and the TIFF files produced were analyzed with Array-Pro Analyzer 6.3 (Media Cybernetics, Rockville, USA). Results were reported as absolute signal intensity by subtracting the array background intensity of the weighted average of the six spots present in the array for a single sample. Since very low binding signals of mABs or CBM were detected for pectic components extracted using CDTA, only signals from 4M NaOH extracts were analyzed. It should be noted that the signals recorded represent a semi-quantitative measure of the polymer considered, relative to the samples analyzed, thus this measure does not represent absolute polymer quantifications. Signal intensities recorded were firstly adjusted based on the weight of initial sample, then signals recorded for the 6 standards included in each array were used to correct for overall signal intensity of each array.

### Phenotype statistical analysis

Due to an observed attack of barley yellow mosaic virus (BaYMV) in the 2009 field trial, which as expected severely affected the traits recorded [25], it was initially decided to keep further analysis separate for the two years. The R package mvngGrAd [26] was used to adjust phenotypic values for field spatial variation based on neighbor plots. The parameters used were as in Lado et al. [27]. Then for grain and straw yield (GYLD and SYLD respectively) a model, described as:

$${\text{y}}_{ik}=\;\mu +{\text{g}}_{i}+e_{ik\;}$$ (1)

was fitted using the lmer function in the lme4 R package [28] where y_ik_ represents the spatial adjusted observation for the i^th^ genotype and the k^th^ replicate, μ represents the intercept, g represents the random effect on the genotype and e_ik_ is the residual term, where e_ik_ ~ N(0,$\sigma_{e}^{2}$) For the phenotypes derived from the CoMPP test, a fixed effect b_j_ was added to account for the batch effect of j^th^ CoMPP extraction. Trait genetic determination (GD) was then calculated for all traits following the formula:

$$GD=\sigma_{g}^{2}/(\sigma_{g}^{2}+\sigma_{e}^{2})$$ (2)

where $\sigma_{g}^{2}$ represents total genetic variance and $\sigma_{e}^{2}$ the residual variance. For each trait and year, the model was used to calculate the genotypes’ best linear unbiased predictor (BLUPs). BLUPs were firstly used to identify significant differences between the two main groups constituting the barley collection, 2- and 6-row type. A simple t-test with default parameters was performed for each trait/year combination using the *t*.*test* function implemented in R. BLUPs were subsequently used to calculate Pearson’s coefficient of correlations between traits within years and within trait between years using the *corr*.*prob* function also implemented in the statistic platform R [29]. For further analysis BLUPs of traits showing correlation between years were recalculated combining data from 2009 and 2010. This was done following the equation

$${\text{y}}_{iktr}=\;T_{r}+{\text{g}}_{i}+gt_{ir}+\;e_{ik}$$ (3)

Where T represents the fixed effect of r^th^ year of trial and tr the genotype by environment interaction of every ir^th^ genotype/year combination. As for Eq (1) an additional fixed effect was added for CoMPP derived phenotypes to account for batch effect. Broad sense heritability of individual measurement (*H*^*2*^) following Leplat et al. [30] was calculated for these traits using the equation:

$$H^{2}=\sigma_{g}^{2}/(\sigma_{g}^{2}+\sigma_{gt}^{2}+\sigma_{e}^{2})$$ (4)

### Genotypic evaluation, population structure and linkage disequilibrium analysis

The DNA extracted from young seedlings and described elsewhere [31] was genotyped with 6810 single nucleotide polymorphism (SNP) markers using the barley iSelect chip based on the Illumina Infinium genotyping assay [2]. To obtain more robust data, markers with more than 10% missing data points were removed, and then the missing genotype data were imputed using the R package scrime [32] based on the five nearest weighted genotypes present in the dataset. Lastly markers with minor allele frequencies (MAF) < 0.05 were excluded. The genetic position of the markers was based on the consensus map recently developed by Munoz-Amatriain et al. [33]. Unmapped markers were included in the analysis when passing filtering for missing data and MAF. STRUCTURE V. 2.3.3 software was used to analyze population structure in the collection [34]. Without any *a- priori* knowledge of the population, each individual is assigned to a subgroup (number of subgroup = *k*) based on multi-locus genotypic data, and the fit of the model is then tested. The software tested k values from 1 to 8 with the entire set of SNP markers, and 10,000 burn-in iterations and 100,000 Markov Chain Monte Carlo (MCMC) iterations were set as parameters for each run. Each value of *k* was tested in 5 replicates. The optimal number of *k* was determined as suggested by Evanno et al. [35] using Structure Harvester (http://taylor0.biology.ucla.edu/structureHarvester/), described in Earl and Vonholdt [36]. Once the number of subgroups was established, Q values provided by STRUCTURE, representing the percentage of membership to each group for each line, were analyzed with the software CLUMPP V. 1.1.2 (http://www.stanford.edu/group/rosenberglab/clumpp.html) as described by Jakobsson and Rosenberg [37] using default settings. Finally, to assign each individual to a subgroup a threshold of Q ≥ 0.7 was assumed to define complete membership otherwise the individual was assigned to a mixed subgroup. In order to verify the results obtained with STRUCTURE, principal component analysis (PCA) was performed on the entire set of markers using the *prcomp* function included in the statistical package R. Scores plots were inspected to verify clustering of the subpopulation. Intra-chromosomal linkage disequilibrium (LD) patterns were also studied using TASSEL v. 3.0.6 [38]. Statistically significant (P < 0.05) mapped pairwise markers r^2^ estimates of LD for each chromosome were calculated and plotted as a function of the distance between the markers being considered. A second-degree smooth LOESS curve was fit on the plotted data-points. To establish an r^2^ threshold value for markers not in LD, the 95^th^ percentile of r^2^ values for unlinked loci (> 50 cM apart) was calculated. The projection onto the x axis of the intercept between the fitted curve and the critical r^2^ value was considered as average distance for LD decay [39]. The same LD analysis was also performed for single chromosomes.

### Genome-wide association mapping and candidate gene selection

Genome wide association mapping was carried out using the package GAPIT [40] implemented in R, to detect positive marker-trait associations (MTAs). Model correction for population structure and cryptic relatedness between lines was based on a compressed EMMA kinship matrix included as random effect. Optimal compression level was obtained by varying the number of groups from 1 to 112 and selecting the correct level based on -2 log likelihood of the fitted model. The package can also include PCs as a fixed effect to correct for population structure and their optimal number is automatically evaluated based on the Bayesian information content (BIC) of the fitted model. Results showed that no improvement was obtained for the model using PCs thus a fixed effect was not included in the final model. GWAS was performed only for the traits showing correlation between year BLUPs. Predictors of genotype performance were derived from Eqs (1) and (3) thus for 2009, 2010 and 2009 plus 2010 (referred as “09+10” hereafter). Results were analyzed to identify significant MTAs and QTLs comparing results from the 3 different GWAS. Markers were considered significant for -log10 (*p)* value > 3. Adjustment of p values for multiple testing was also considered using the false discovery rate (FDR) method implemented in GAPIT. Yet, given the number of genotypes included and the low heritability of traits as those involving cell wall components [41, 42] it was decided not to systematically exclude MTAs failing at FDR p value adjustment but to evaluate every MTAs within its context, thus considering: *1)* how many markers resulted significant from the same or different analysis mapping at similar positions; *2)* presence of known genes regulating the trait considered in the region; *3)* SNP allele frequency.

Markers that were shown to be significant but were not mapped according to Munoz-Amatriain et al. [33] were assigned to a genomic position using the on-line tool Barleymap (http://floresta.eead.csic.es/barleymap/) applying marker name as search criterion. The database was used to retrieve lists of candidate genes in the genomic regions identified by the significant markers (for barley genome, genes are coded as MLOCs). These lists were obtained by searching in the database for genes between markers. The boundaries of the regions searched were determined based on the calculated average LD decay for the chromosome of interest. Where a clear candidate gene was not identified, gene expression profiles retrieved from the data generated by the barley physical map annotation project [1] were analyzed for the developing tillers at six-leaf stage. The list of genes was restricted considering only expressed genes with transcriptional level (FPKM) > 1. Gene annotation of the selected genes was then analyzed for possible candidate genes.

### Candidate gene analysis

GWAS results for GYLD allowed further analysis of the identified candidate gene, Hv-eIF4E [43]. The entire winter barley collection was genotyped for the *rym4* and *rym5* allelic state at the considered gene by using CAPS markers following the procedure of Sedlacek et al. [44]. The results were used to verify the co-segregation degree between iSelect markers identified as significant for GYLD and the CAPS markers screened. Furthermore, GWAS was performed a second time including these markers. The genetic position in cM for Hv-eIF4E was retrieved from the Barley Floresta database (http://Floresta.eead.csic.es/barleymap/).

## Results

### Field trial

The raw phenotypic data were analyzed in the first instance to identify trait variation between the years. Descriptive statistics of the trait recorded are given in Table 1.

**Table 1:** Descriptive statistics for field trials.

	2009				2010				*H*^*2*^^c^
Trait	min	mean	max	GD^b^	min	mean	max	GD^b^	
GYLD (t/ha)	1.35	3.28	6.59	0.49	2.74	6.82	10.69	0.70	0.42
SYLD (t/ha)	3.09	5.86	10.56	0.09	4.56	11.54	21.93	0.29	-
LM6 ^a^	20.05	27.76	40.39	0.53	15.16	21.37	34.09	0.27	0.32
BS-400-3 ^a^	47.33	55.27	67.44	0.16	39.48	48.29	56.58	0.22	-
JIM13 ^a^	14.84	18.87	22.8	0.17	12.91	18.32	25.04	0.12	0.20
LM11 ^a^	49.64	61.34	72.95	0.18	50.49	58.95	68.39	-	-
CBM3a ^a^	12.71	16.55	21.91	-	13.77	16.77	21.08	0.04	-

Summary of phenotypic data recorded, calculated genetic determination (GD), and broad sense heritability of single measurement (*H*^*2*^*)*.

^a^ adimensional measure of binding signal intensity, see monoclonal antibody and carbohydrate binding module specificity in materials and methods.

^b^ Genetic determination calculated as per Eq (2).

^c^ Broad sense single measurement heritability was calculated only for traits where a correlation was identified between BLUPs from different year analysis.

Mean values for GYLD and SYLD for the first year of trial were around 50% less than those in the second year (48.1% and 50.8%, respectively). The opposite results were recorded for traits related to culm cell wall components where all traits, except CBM3a, showed lower mean values in 2010 compared with 2009. LM6 and BS-400-3 in particular showed a pronounced reduction (87.4% and 77% respectively). GD in both years was relatively high for GYLD compared to the other traits, except for LM6 in 2009 (Table 1). SYLD showed low GD values for both years, especially in 2009 (GD = 0.09) while it was higher in 2010 (GD = 0.29). LM6 GD was equal to 0.53 for 2009 while it showed a lower value in 2010 (GD = 0.27). Most of the remaining GD values varied between 0.16 and 0.22, except for CBM3a 2010 (GD = 0.04) and for LM11 2010 and CBM3a 2009 where no genetic effect was detected. Thus, BLUPs were not calculated for these two traits. The t-test performed to identify phenotypic differences between 2- and 6-row genotypes showed that for LM6 in both years 2-row types had significantly higher values (p value < 0.001). BS-400-3 for 2009, GYLD and JIM13 for 2010 also resulted to be significantly different between the row types although to a lesser extent (p value < 0.05). See data in S3 Table for details. A study of Pearson’s correlations of BLUPs is reported in Table 2. A correlation between traits within each year showed a consistent positive correlation in both years between GYLD and SYLD and between JIM13 and LM6. A negative correlation was detected between GYLD and LM6 in both field trials. For the same trait between years, correlation results showed that GYLD and LM6 were the traits with highest values (0.69*** and 0.46*** respectively) followed by JIM13 (0.40***). No significant (P <0.001) correlation was found for the remaining traits and they were therefore omitted from further analysis. When considering *H*^*2*^ values GYLD showed relatively high heritability with 0.42 followed by LM6 (0.32) and JIM13 (0.20).

**Table 2:** BLUPS correlations.

		2009								
		GYLD	SYLD	LM6	BS-400-3	JIM13	LM11	CBM3a		
2010	GYLD	0.69***	0.43***	-0.49***	n.s.	-0.39***	n.s.	n.a.	GYLD	2009
	SYLD	0.53***	n.s.	-0.40***	n.s.	n.s.	n.s.	n.a.	SYLD	
	LM6	-0.39***	n.s.	0.48***	n.s.	0.53***	n.s.	n.a.	LM6	
	BS-400-3	n.s.	n.s.	0.56***	n.s.	n.s.	0.37***	n.a.	BS-400-3	
	JIM13	n.s.	n.s.	0.40***	0.34***	0.40***	n.s.	n.a.	JIM13	
	LM11	n.a.	n.a.	n.a.	n.a.	n.a.	n.s.	n.a.	LM11	
	CBM3a	n.s.	n.s.	n.s.	n.s.	n.s.	n.s.	n.a.	CBM3a	
		GYLD	SYLD	LM6	BS-400-3	JIM13	LM11	CBM3a		
		2010								

Pearson’s correlation between trait BLUPS calculated for trial 2009 (upper triangle) and 2010 (lower triangle).

***: significant correlation values at p <0.001. Grey cells: correlations for the same trait between years. Green cells: significant correlation detected both years independently for two different traits. n.s.: not significant; n.a.: not available.

### Molecular markers, population structure and LD

**Fig 1:**
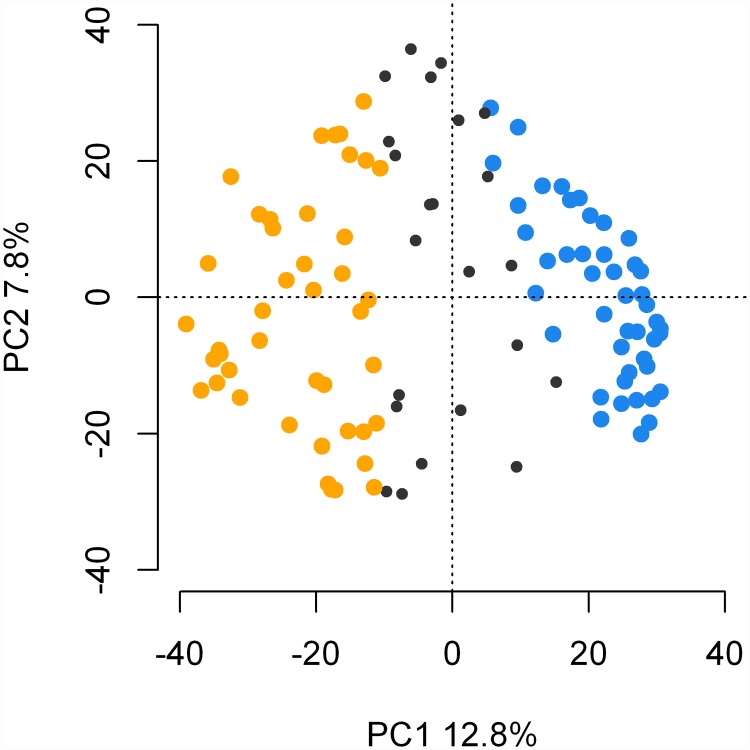
Population structure analysis. Scores plot of PC1 vs. PC2 from PCA on the markers analyzed. Blue and yellow colors correspond to 6-row and 2-row groups respectively as assigned based on results from STRUCTURE. Branches and closed circles in black color correspond to genotypes not uniquely assigned to a group.

After filtering the markers, a total of 4976 SNPS were used for subsequent analysis. Of these, 1,284 were recorded as unmapped based on the consensus map used for the present study. Population structure analysis using STRUCTURE and PCA identified two subpopulations according to spike morphology (two-row and six-row type). Based on q values obtained from STRUCTURE and CLUMPP, 44 varieties were assigned to the two-row group, 45 to the six-row group and 23 were noted as admixed. Results are given in Fig 1.

**Fig 2:**
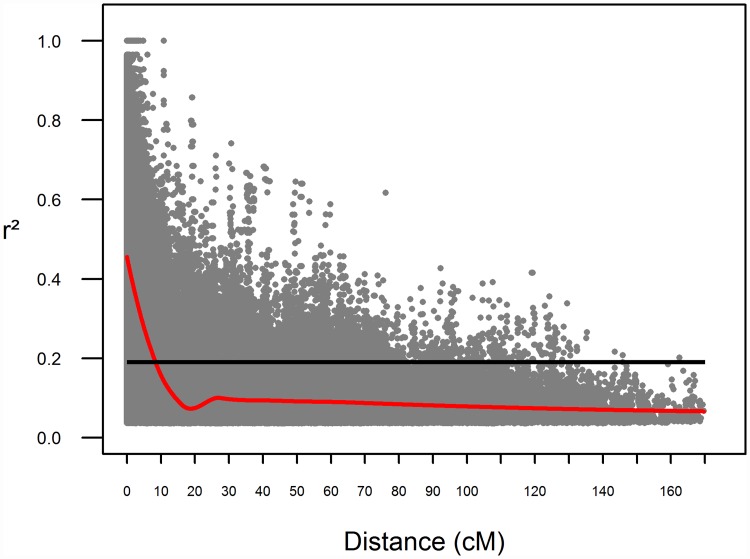
Average intra-chromosomal LD decay. r^2^ values of LD are plotted as a function of the distance between pairs of markers considered. Black line: r^2^ values of the 95^th^ percentile for unlinked (>50 cM) markers. Red line: second degree smoothed loess curve.

An analysis of average intra-chromosomal LD decay was performed based on a total of 493,638 significant pairwise marker comparisons. Of these, 104,748 were markers more than 50 cM apart, and thus were used to calculate an LD threshold to consider whether markers were in LD or not. The LD threshold was found to be r^2^ = 0.19 and the fitted smoothed loess curve crossed the threshold at 7.96 cM (Fig 2). When LD decay was analyzed for each chromosome, 7H was found to show the more extended LD (11.24 cM) followed by 5H (10.73 cM) and 2H (9.17 cM). Chromosome 6H instead was the one showing the most rapid LD decay with an average of 3.98 cM. The remaining chromosomes 1H, 3H and 4H showed an LD extent of 8.60, 8.23 and 4.76 cM respectively.

### Genome-wide association mapping and candidate genes

**Fig 3:**
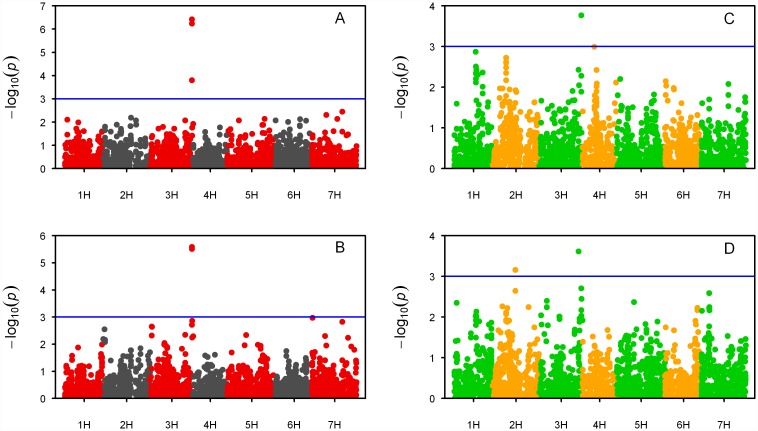
Manhattan plots from GWAS results. GWA scan results for A) GYLD 2009; B) GYLD 2010; C) LM6 2009; D) LM6 2010. The blue horizontal line in each plot represents the arbitrary significant threshold at -log10 (p value) = 3.

Three traits were analyzed for marker-trait association: GYLD, LM6 and JIM13. These were the traits showing significant correlation of BLUPs between the two years. The results are summarized in Table 3 and presented in Fig 3 and S1 Fig. A total of 28 significant (-log10(*p*) > 3) associations were identified for single year BLUPS and 15 for combined years analysis. Results from GYLD highlighted one single QTL on 3H at ~ 160 cM for each year and for 09+10, with 3 MTAs present for all analysis in the same region (Table 3, Fig 3A and 3B). Such 3 MTAs were also the only significant when considering FDR adjusted *p* values (< 0.05). When considering the most significant marker for the QTL (SNP ID: 11_10767), the 43 varieties possessing the less frequent allele [A] yielded on average 0.52 and 1.45 t/ha more in 2009 and 2010, respectively than the 69 varieties carrying the more represented allele. These high yielding varieties were all released after 1980 except for the six-row varieties ‘Dea’ and ‘Dura’ released in Germany in 1953 and 1960 respectively. No particular pattern according to year of release was observed for genotypes possessing the major allele. It is worth noting also that alleles were roughly equally spread between two-row and six-row types. A search for relevant markers for GYLD in both years was conducted in the Floresta Barley Map database. The identified QTL region spanned ~7 Mbp between 557 and 564 Mbp. The barley ‘eukaryotic translation initiation factor 4E’ (*Hv-*eIF4E) was present in the region (MLOC_4680) at 556,890,556 bp on 3H (data retrieved from: http://plants.ensembl.org/Hordeum_vulgare), and was thus tightly linked to the QTL identified for GYLD. *Hv-eIF4E* is a gene known to regulate resistance to BaYMV, and two alleles—*rym4* and *rym5*—have so far been reported for the gene conferring resistance to a different virus strain [43, 45, 46]. Given the reported presence of BaYMV in field trials in 2009, *Hv-eIF4E* would appear to be a solid candidate gene regulating GYLD in the study presented here.

**Table 3:** GWAS results.

Trait	Year	Marker ID	Chr.	Pos. cM	-log_10_(p)^#^	R^2^	MAF	Floresta Chr^$^	Floresta Pos cM^$^
GYLD	2009	11_10767	3	160.08	6.42**	0.23	0.38	3	154.16
		11_11516	3	159.55	6.24**	0.22	0.36	-	-
		SCRI_RS_146798	-	-	4.87*	0.16	0.35	3	148.58
		SCRI_RS_236603	3	159.99	3.80	0.12	0.36	3	154.82
		SCRI_RS_143505	3	160.09	3.80	0.12	0.36	3	155.03
		SCRI_RS_160338	3	160.09	3.80	0.12	0.36	3	155.03
		SCRI_RS_178836	3	160.09	3.80	0.12	0.36	3	155.03
		SCRI_RS_184261	3	160.09	3.80	0.12	0.36	3	155.03
		SCRI_RS_237738	3	160.09	3.80	0.12	0.36	3	155.03
	2010	11_10767	3	160.08	5.59**	0.17	0.38	3	154.16
		11_11516	3	159.55	5.52**	0.16	0.36	-	-
		SCRI_RS_146798	-	-	5.03*	0.15	0.35	3	148.58
		SCRI_RS_159189	-	-	3.18	0.08	0.13	3	155.03
		SCRI_RS_162720	-	-	3.18	0.08	0.13	3	155.03
	09+10	11_10767	3	160.08	6.74***	0.21	0.38	3	154.16
		11_11516	3	159.55	6.29**	0.19	0.36	-	-
		SCRI_RS_146798	-	-	5.31**	0.15	0.35	3	148.58
		SCRI_RS_7217	7	114.1	3.23	0.08	0.18	7	102.94
LM6	2009	SCRI_RS_236603	3	159.99	3.77	0.11	0.36	3	154.82
		SCRI_RS_143505	3	160.09	3.77	0.11	0.36	3	155.03
		SCRI_RS_160338	3	160.09	3.77	0.11	0.36	3	155.03
		SCRI_RS_178836	3	160.09	3.77	0.11	0.36	3	155.03
		SCRI_RS_184261	3	160.09	3.77	0.11	0.36	3	155.03
		SCRI_RS_237738	3	160.09	3.77	0.11	0.36	3	155.03
	2010	12_30960	3	149.06	3.62	0.12	0.08	3	143.13
		SCRI_RS_130177	3	149.06	3.62	0.12	0.08	3	143.13
		SCRI_RS_141898	-	-	3.62	0.12	0.08	3	143.13
		11_20781	2	88.04	3.16	0.10	0.15	2	76.7
		SCRI_RS_133327	-	-	3.16	0.10	0.15	2	80.03
		SCRI_RS_154203	-	-	3.16	0.10	0.15	-	-
		SCRI_RS_181300	-	-	3.10	0.10	0.39	1	83.71
	09+10	SCRI_RS_120182	-	-	3.80	0.10	0.05	2	38.95
		SCRI_RS_181300	-	-	3.62	0.10	0.39	1	83.71
		SCRI_RS_236603	3	159.99	3.60	0.10	0.36	3	154.82
		SCRI_RS_143505	3	160.09	3.60	0.10	0.36	3	155.03
		SCRI_RS_160338	3	160.09	3.60	0.10	0.36	3	155.03
		SCRI_RS_178836	3	160.09	3.60	0.10	0.36	3	155.03
		SCRI_RS_184261	3	160.09	3.60	0.10	0.36	3	155.03
		SCRI_RS_237738	3	160.09	3.60	0.10	0.36	3	155.03
		SCRI_RS_9158	1	82.45	3.05	0.08	0.39	1	81.02
JIM13	2010	11_21398	3	8.86	3.30	0.12	0.24	-	-
	09+10	SCRI_RS_139793	-		3.48	0.12	0.13	2	49.50
		SCRI_RS_181300	-		3.07	0.10	0.39	1	83.71

GWAS results reported for the traits where markers above the arbitrary threshold of (-log10(*p*) > 3) were detected.

^#^ Level of significance for FDR adjusted p values reported along with p values in the column:

*** < 0.001,

** < 0.01,

* < 0.05.

^$^ Data obtained from Barley Map Floresta database (http://Floresta.eead.csic.es/barleymap/)

The trait JIM13 did not show any MTA in 2009 while for 2010 one marker appeared to be significant on 3H at 8.86 cM. GWAS for 2009 and 2010 combined resulted in 2 MTAs on 1H and 2H according to FLORESTA database. However, given the inconsistencies between resultsfrom different GWAS, no further analysis of this trait were carried out.

LM6, the mAB used to detect α (1→ 5)-arabinans in the hemicellulosic component of culms showed six MTAs at ~160 cM on 3H encompassing a single QTL for GWAs of 2009 (Table 3, Fig 3C). These 6 important SNPs were among those found significant for GYLD 2009. Results for the 2010 analysis yielded 7 MTAs (Table 3, Fig 3D). Two of them mapped at 149.06 cM on 3H, and thus did not coincide with the results of the previous year, and one on 2H at 88.04 cM, while the four remaining SNPs were unmapped. When GWAS was performed on BLUPs from 2009 and 2010, 9 MTAs were identified. Among them, the 6 identified on 3H from 2009 analysis were present, as well as SCRI_RS_181300 located on 1H identified in the 2010 analysis. An additional association on 1H resulted significant above chosen level of -log10 p > 3. Finally, SCRI_RS_120182 on 2H resulted as the most significant association for the analysis (-log10 *p* = 3.8). It has to be noted however that such single SNP possessed very low MAF (0.05) thus did not appeared to constitute a reliable association worth to investigate further. When a search for the SNPs in the Floresta database was undertaken, three of the four unmapped markers were assigned to a genetic position (see Table 3). It should be noted that the Floresta database uses a different genetic map to assign marker positions, thus the positions did not completely correspond with the consensus map used here. Nevertheless, the differences were relatively small and did not exceed 11 cM for marker 11_20781 (88.04 cM in consensus map, 76.7 in Floresta database).

It is also worth to mention that no MTAs resulted significant (p < 0. 05) after adjustment of p values for FDR. Yet, given the criteria previously expressed (see Material and methods section) it was decided to further investigate the region identified on 3H.

Allele frequencies for the six markers located at ~160 cM on 3H did not show any specific pattern according to row type. 6-row varieties showed approximately equal distribution (MAF = 0.49) of alleles while 2-row varieties showed relatively higher frequencies for the major allele (MAF = 0.32, see S1 Table for details). The study of the allelic effects showed how QTLs found on 3H for both years had the highest influence on LM6 with varieties possessing the minor allele for the most significant marker showing an increased level of LM6 of 4.8 and 6.1% for 2009 (SNP ID: SCRI_RS_236603) and 2010 (SNP ID: 12_30960) respectively. For GWAs of LM6 2009, the retrieved list of genes was constituted by 101 high confidence genes spanning a genomic region of ~11 Mbp between 553 and 564 Mbp. For 2010 the QTL on 3H encompassed 218 high confidence genes located in the region between 537 Mbp and 557 Mbp. In total 319 genes, 46 in common between QTL 2009 and 2010, were initially considered. The list was then reduced to 176 genes, removing those not expressed (pkfm < 1) in developing tillers as reported from the barley physical map annotation project. 35 genes were present in both years. 38 were present only for GWAs in 2009 and 103 only in 2010. Gene annotation was analyzed to identify genes involved in carbohydrate biosynthesis. One gene (MLOC_57524) common to QTL-2009 and QTL-2010 was annotated as Beta-fructofuranosydase, (GH 32–43). Furthermore, for QTL-2010 alone 5 genes involved in carbohydrate metabolism were identified: MLOC_11452 (putative beta-1,3-glucanase, GH 17); MLOC_12571 (Beta-1,3-glucanase, GH17); MLOC_19196 (Alpha-1,3-mannosyl-glycoprotein beta-1,2-N-acetylglucosaminyltransferase, GH13); MLOC_ 53950 (Cellulose synthase-like A1, GT 2); MLOC_68021 (Beta-galactosidase, GH 2). These 5 genes appeared to be the major candidates according to gene annotation related to culms cell wall composition.

### Grain yield candidate gene analysis

Genotyping of the winter barley collection with markers for *rym4* and *rym5* alleles was successful except for three genotypes: ‘Naomie’, ‘Opal’ and ‘Panda’ due to technical issues (see S4 Table for details). Scores from the remaining 109 lines showed a tight linkage between *rym4* alleles and the three most significant SNPs associated with GYLD (95% for 11_10767, 92% for 11_11516 and 93% for SCRI_RS_146798). In contrast, the resistance allele for *rym5* was not present in the collection except in the variety ‘Saigon’. When GWAS was performed a second time to include marker scores for *rym4* and *rym5*, only *rym4* resulted in a highly significant association in both years (-log10(*p*) values of 4.4 and 4.9 for 2009 and 2010 respectively).

## Discussion

### Field trial and GWAs for grain yield

A collection of winter barley varieties released in the last century in several European countries was tested in a Mediterranean environment for two years of field trials. Trait GD and correlation of GYLD between the two years was high, confirming a strong genetic influence on the final production of grain biomass. There has been wide-spread coverage in literature of the progress made in the last century in breeding for a high and stable yield [47, 48], and the introduction of favorable alleles in the most recent winter barley varieties clearly had showed a beneficial impact in the results presented here too. The favorable allele for the QTL detected on 3H un the study of grain yield was present in 43 accessions and only 2 of these lines were released before 1980. Notably, the first year of the trial saw a strong incidence of BaYMV, which affected the final grain yield. BaYMV is a soil-borne virus belonging to the *Bymovirus* genus in the *Potyviridae* family. It is transmitted by plasmodiophorid *Polymyxa graminis*. Since the vector can be found down to a soil depth of 60 cm treatment against it is not agronomically viable [43]. The virus is of major importance in winter barley causing yield losses of up to 50%. Given the highly reduced grain yield observed, decision was taken to perform GWA mapping separately for the two years. This allowed verification of whether the contrasting grain yield results were due to different genetic effects occurring in the trials, thus expecting different QTLs to be found when GWA was performed, or whether a stable effect was observed due to a single QTL present in both years. The results confirmed the second hypothesis to be correct finding a QTL at the telomeric region of 3HL. For GYLD in both years it was hypothesized that the QTL found was due to the presence of resistance genes to the virus BaYMV, identifying Hv-eIF4E as being relatively close to the QTL region. Interestingly, the resistance allele also appeared to be effective in the second year of the trial when no virus incidence was detected. It should be noted that the field trials where physically separate from one another each year, thus it was speculated that different growing condition may have exacerbated the virus attack in the first year of the trial or that the presence of different levels of the virus vector dramatically impacted the spread of the virus. However, since the presence of the virus was not detailed during the trials, definitive conclusions cannot be drawn or correlated with yield performance. Given that the same QTL for resistance to BaYMV was found in both years, it is possible that the virus was also present in both years although with no detectable incidence. It is known that resistance genes have been introduced in European winter barley varieties, mostly the *rym4* and *rym5* alleles, initially thought to be distinct, closely located genes but subsequently found to be functional variants of the same resistance factor Hv-eIF4E at different exons in the gene [43]. More resistance genes have been identified and are known to be spread throughout the barley genome, e.g. rym11 on 4HL [46], Rym17 on 3H and rym18 on 4H [49]. Most of the European varieties carry the rym4 resistance allele, first introduced in Germany in the 1980s but overcome shortly afterwards by a new strain of the virus. Varieties carrying rym5 were first released in the late1990’s, e.g. ‘Saigon’ in 2002. These are resistant to both BaYMV and a new strain identified as BaYMV-2 [50].

Given that the barley collection investigated here included varieties released more than seventy years ago, the possibility was investigated that the favorable QTL observed was due to the presence of semi-dwarfing genes conferring high yield. The *sdw1/denso* semi-dwarfing gene in barley is one of the so-called “green revolution” genes introduced in cereal crops such as maize and rice conferring reduced plant height, which is necessary for modern intensive agriculture [51]. In barley, Malosetti et al. [52] mapped the gene at 127.1 cM on 3H using BOPA2 marker 12_30096, which was included in the present GWAs but did not show any significance. To further support this, the CDs sequence of the *sd1* gene, the rice orthologue of *Sdw1* [53] was retrieved from the Rice Genome Annotation Project database (http://rice.plantbiology.msu.edu/index.shtml, LOC_Os01g66100) and blasted against the barley genome in the Ensemble database (http://plants.ensembl.org/Hordeum_vulgare/). The results showed that the best hit was located on MLOC_56462 on 3H at 509 MBp, approximately 50 MBp away from the QTL identified in this study, further excluding an involvement of semi-dwarfing genes in the yield performances observed here. rym4/rym5 resistance alleles for Hv-eIF4E have been extensively studied and several markers have been developed to rapidly screen lines for marker assisted selection (MAS) in breeding programs. After the gene was cloned, SNPs diagnostics for the two alleles were identified [43]. Moreover, simple, fast and cost-effective cleaved amplified polymorphic sequence (CAPS) markers are now available [44] to screen for lines possessing resistance alleles and the simple sequence repeat (SSR) marker QLB1 has also been developed [54]. However, the barley 9K iSelect genotypic platform is currently one of the most frequently used to genotype barley lines in QTL mapping studies, breeding and genomic selection programs. The platform is well established [2] and the reduced costs coupled with the possibility of simultaneously screening the whole barley genome currently make it the most versatile tool for detecting causal genes for agronomically relevant traits [55, 56]. The iSelect marker 11_10767 detected here appeared to be tightly linked with the gene Hv-eIF4E and the presence of the resistance allele conferred a higher yield compared to genotypes carrying the non-resistance allele. Since iSelect markers are based on expressed sequence tags (ESTs) it is possible to verify whether some of the SNPs used in this study were present directly on Hv-eIF4E. SNPs are reported as unigenes in the HarvEST barley database (version 1.83, assembly 35, http://harvest-web.org/hweb/hmain.wc?versid=5) and the best hit for Hv-eIF4E was on unigene U2412. None of the available iSelect markers is located on such a unigene. Subsequently, physical distance between the candidate gene and the most significant marker, SNP 11_10767 was identified as being derived from unigene U35_3081 (source: http://bioinf.hutton.ac.uk/iselect/app/). The HarvEST barley database (version 1.83, assembly 35) was interrogated again and retrieve unigene sequence retrieved, which was subsequently blasted against the barley genome in the Ensemble database (ttp://plants.ensembl.org/Hordeum_vulgare/). The best hit (ID% 99.8, E-val: 0.0) proved to be on MLOC_115, located at 557,826,427 bp on 3H, less than 1 Mbp from Hv-eIF4E, 35 MLOCs were included in the region separating the marker from the candidate gene. Although this number of MLOCs is relevant as an absolute value, when considering the relatively high extent of barley LD for chromosome 3H (8.23 cM), Hv-eIF4E can still be considered the strongest candidate gene for the QTL identified from studying GYLD. Analysis of the segregation between rym4 and rym5 diagnostic markers and the iSelect SNPs defining the QTL showed strong, although not complete, linkage. As a consequence, it is not possible to consider the iSelect marker 11_10767 as a fully diagnostic tool to discern resistant lines to BaYMV carrying the *rym4* allele from susceptible ones. Nevertheless, given that at present the great majority of QTL mapping and breeding programs employ the iSelect array to genotype lines, the results reported here could offer additional support in the process of selection of breeding material which may require further study in the case of a specific focus on BaYMV resistance.

### Culms cell wall composition

To characterize the culm cell wall of the collection of winter barley varieties considered here, the CoMPP test was employed. To the authors’ knowledge, this is the first time that such a test has been employed in a GWA study. The CoMPP test is a robust technique to study plant cell wall components and it has been successfully applied to a number of different plant species as well as algae for studies on the evolution of species, biofuel production and plant physiology [57–59]. This immune detection method, which is high-throughput in terms of the number of samples extractable in a single run and is coupled with the possibility of screening for a wide range of cell wall polysaccharide epitopes, makes it an efficient tool for investigating complex traits such as plant cell walls. In the study presented here a general trend of low genetic influence was observed. In two cases, no genetic effect was detected (LM11- 2010 and CBM3a—2009). Plants are known to possess properties of plasticity, allowing cell wall composition to be varied according to the biotic and abiotic stresses that can occur, making genetic effects hard to detect especially in non-controlled growing conditions. Interestingly the trait LM6 showed the highest GD values among plant cell wall components, in particular in the first year of trial, which made this trait promising for a GWA study. Remarkably, LM6 signal appeared to be higher in culm cell wall derived from 2-row genotypes in both years of trials despite the different growing conditions occurred. To the authors knowledge this represents a novelty that will require further studies to confirm and possibly explain.

Prior to formulating a hypothesis about putative candidate genes involved in the regulation of the epitopes recognized by LM6, various considerations need to be taken into account. In fact, the antibody LM6 is known to possess specificity to bind α (1→ 5)-arabinan epitopes present in pectic polysaccharides (http://www.ccrc.uga.edu/~mao/wallmab/Antibodies/antib.htm). However, the plant material studied here was barley culms collected at grain maturity where the majority of cell wall polysaccharides are constituted by cellulose and hemicellulose (mainly heteroxylan backbones carrying arabinofuranose residues at C_2_ and C_3_ position). In this case, if present at all pectin is supposed to be found in trace amounts [60]. Moreover, signals derived from the CDTA extraction, which specifically extract pectins, were observed. Therefore, it was speculated that the mAB LM6 was binding to different polysaccharides present in the samples rather than pectic arabinose. Specifically, the possibility that the mAB LM6 signals were derived from the arabinose side chain in arabinogalactan proteins (AGPs) and hemicellulosic arabinose residues was evaluated. AGPs can contain α (1→ 5)-arabinans [61] and mAB LM6 is reported to show cross-reactivity with them (see http://glycomics.ccrc.uga.edu/wall2/jsp/abIndex.jsp). This would explain the positive correlation found between LM6 and JIM13 BLUPs in both years of this study. AGPs are a highly diverse class of cell surface glycoproteins involved in a wide range of mechanisms, such as reproduction, cell proliferation and abiotic stress response [62] and are known to be present in most plant species. In barley their role has been highlighted with regard to root hair development [63] and it has been suggested that they are involved in non-host resistance signaling [64]. Cross-reactivity of LM6 with arabinoxylans is also reported. However, if this were the case here, a correlation between LM6 and LM11 (specific for xylans / arabinoxylans) would be expected, However, no such result was observed. It was not possible to verify whether LM6 binding signals were derived from arabinogalactan proteins or hemicellulosic arabinoxylans, but AGP appeared more likely due to the observed positive correlation between LM6 and JIM13 signal BLUPs. Interestingly a negative correlation between GYLD and LM6 was observed and to the authors’ knowledge is a new finding that has not yet been reported. As discussed above, in the field trials GYLD was related to resistance to BaYMV thus an involvement of cell wall epitopes bound by mAB LM6 and cross-reacting with AGPs in such resistance seems a reasonable hypothesis. However, knowledge about AGPs and their involvement in response to biotic stresses is poorly documented. Zhang et al. [65] recently investigated xylem sap protein content in cotton identifying several fasciclin-like AGPs involved in cell wall metabolism and development as well as disease resistance. More investigations are required to verify if and how AGPs regulates plant response to biotic stresses and how they are involved in the variation of agronomically relevant traits.

### GWAs for LM6 and outlook

As pointed out by Alexandersson et al. [66], the need to link data from field experiments to networks of genes regulating complex traits is of primary importance. While knowledge about plant cell wall composition, regulation and function mostly originates from laboratory-scale experiments, it is relevant to verify the results at the field scale. Examples of the difficulties encountered in doing this are available. Phenotypic platforms to efficiently monitor growing conditions are under development in an attempt to close the gap between the laboratory and the field [67, 68], but the complete range of environmental stresses during plant growth in the field is hard to mimic in controlled environments. In consideration of this, an attempt has been presented to characterize a collection of winter barley varieties for plant cell wall related traits and identified QTLs underlying causal genes responsible for the phenotypic variation observed in the field. Epitopes recognized by the mAB LM6 were the ones showing the highest trait GD and *H*^*2*^ as well as the ones with the highest correlation between BLUPs in the two years of study. For the 2009 and combined 09+10 trials a region on 3H was identified at ~154.8 cM, while in 2010 the 3 most significant MTAs were still on 3H but at 143.13 cM. Given the strong variation in growth condition between the two years it is possible that this could be the reason for the slight difference in location of the identified QTL between the years. It has to be remembered however that all the MTAs underlying this QTL appeared not significant when p values were corrected for multiple testing. This was somehow expected given the number of lines included in the study and the complexity of the trait likely to be regulated by many loci each contributing small effects. In these cases FDR may result too stringent and for this reason it was decided to include additional analysis on the QTL [41, 69]. Searching for candidate gene post GWAs is a complex task, especially when the traits studied are not well known and are characterized as arabinans and AGPs. The data generated form this study and the bioinformatics resources available online highlighted six candidate genes, one of them present in both years of the analysis, expressed in culms involved in carbohydrate metabolism. In future, with the fast-paced growth in genomic resources for barley, an increased amount of information regarding gene function and expression will help to better characterize the region on 3H of interest for arabinans/AGPs.

## Supporting information

S1 Fig Manhattan and QQ plots for GWAS on all traits and datsets. (PDF)

S1 Table List of winter barley varieties included in the study and genotypic information. (XLSX)

S2 Table Raw phenotypic data. (XLSX)

S3 Table Summary of BLUPs values divided by row type and results of t-test to identify differences between the 2 groups. (XLSX)

S4 Table Allelic state of rym4 and rym5 for the population studied screened with CAPS markers. (XLSX)

## References

[1] Mayer KFX, Waugh R, Langridge P, Close TJ, Wise RP, Graner A, et al A physical, genetic and functional sequence assembly of the barley genome. Nature. 2012;491(7426):711-+ 10.1038/nature11543 23075845

[2] Comadran J, Kilian B, Russell J, Ramsay L, Stein N, Ganal M, et al Natural variation in a homolog of Antirrhinum CENTRORADIALIS contributed to spring growth habit and environmental adaptation in cultivated barley. Nat Genet. 2012;44(12):1388–92. 10.1038/ng.2447 23160098

[3] Ramsay L, Comadran J, Druka A, Marshall DF, Thomas WTB, Macaulay M, et al INTERMEDIUM-C, a modifier of lateral spikelet fertility in barley, is an ortholog of the maize domestication gene TEOSINTE BRANCHED 1. Nat Genet. 2011;43(2):169–U25. 10.1038/ng.745 21217754

[4] Visioni A, Tondelli A, Francia E, Pswarayi A, Malosetti M, Russell J, et al Genome-wide association mapping of frost tolerance in barley (Hordeum vulgare L.). BMC Genomics. 2013;14. 10.1186/1471-2164-14-424 PMC3701572 23802597

[5] Matthies IE, Malosetti M, Roder MS, van Eeuwijk F. Genome-Wide Association Mapping for Kernel and Malting Quality Traits Using Historical European Barley Records. PLoS One. 2014;9(11). 10.1371/journal.pone.0110046 PMC4221631 25372869

[6] Burton RA, Fincher GB. Current challenges in cell wall biology in the cereals and grasses. Frontiers in Plant Science. 2012;3. 10.3389/fpls.2012.00130 PMC3375588 22715340

[7] Yang F, Mitra P, Zhang L, Prak L, Verhertbruggen Y, Kim JS, et al Engineering secondary cell wall deposition in plants. Plant Biotechnol J. 2013;11(3):325–35. 10.1111/pbi.12016 23140549 PMC3644865

[8] Malinovsky FG, Fangel JU, Willats WGT. The role of the cell wall in plant immunity. Frontiers in Plant Science. 2014;5. 10.3389/fpls.2014.00178 PMC4018530 24834069

[9] McCann MC, Carpita NC. Biomass recalcitrance: a multi-scale, multi-factor, and conversion-specific property. J Exp Bot. 2015;66(14):4109–18. 10.1093/jxb/erv267 26060266

[10] Scheller HV, Ulvskov P. Hemicelluloses. Annual Review of Plant Biology, Vol 61. 2010;61:263–89. 10.1146/annurev-arplant-042809-112315 20192742

[11] Vogel J. Unique aspects of the grass cell wall. Curr Opin Plant Biol. 2008;11(3):301–7. 10.1016/j.pbi.2008.03.002 18434239

[12] Liepman AH, Wightman R, Geshi N, Turner SR, Scheller HV. Arabidopsis—a powerful model system for plant cell wall research. Plant J. 2010;61(6):1107–21. 10.1111/j.1365-313X.2010.04161.x 20409281

[13] Schwerdt JG, MacKenzie K, Wright F, Oehme D, Wagner JM, Harvey AJ, et al Evolutionary Dynamics of the Cellulose Synthase Gene Superfamily in Grasses. Plant Physiol. 2015;168(3):968–83. 10.1104/pp.15.00140 25999407 PMC4741346

[14] Nakano Y, Yamaguchiz M, Endo H, Rejab NA, Ohtani M. NAC-MYB-based transcriptional regulation of secondary cell wall biosynthesis in land plants. Frontiers in Plant Science. 2015;6:18. 25999964 10.3389/fpls.2015.00288 PMC4419676

[15] Taylor-Teeples M, Lin L, de Lucas M, Turco G, Toal TW, Gaudinier A, et al An Arabidopsis gene regulatory network for secondary cell wall synthesis. Nature. 2015;517(7536):571–5. 10.1038/nature14099 25533953 PMC4333722

[16] Penning BW, Sykes RW, Babcock NC, Dugard CK, Held MA, Klimek JF, et al Genetic Determinants for Enzymatic Digestion of Lignocellulosic Biomass Are Independent of Those for Lignin Abundance in a Maize Recombinant Inbred Population. Plant Physiol. 2014;165(4):1475–87. 10.1104/pp.114.242446 24972714 PMC4119032

[17] Bellucci A, Torp AM, Bruun S, Magid J, Andersen SB, Rasmussen SK. Association Mapping in Scandinavian Winter Wheat for Yield, Plant Height, and Traits Important for Second-Generation Bioethanol Production. Frontiers in Plant Science. 2015;6. 10.3389/fpls.2015.01046 PMC4660856 26635859

[18] Slavov GT, Nipper R, Robson P, Farrar K, Allison GG, Bosch M, et al Genome-wide association studies and prediction of 17 traits related to phenology, biomass and cell wall composition in the energy grass Miscanthus sinensis. New Phytol. 2014;201(4):1227–39. 10.1111/nph.12621 24308815 PMC4284002

[19] Ramstein GP, Lipka AE, Lu F, Costich DE, Cherney JH, Buckler ES, et al Genome-Wide Association Study Based on Multiple Imputation with Low-Depth Sequencing Data: Application to Biofuel Traits in Reed Canarygrass. G3-Genes Genom Genet. 2015;5(5):891–909. 10.1534/g3.115.017533 PMC4426374 25770100

[20] Mocoeur A, Zhang YM, Liu ZQ, Shen X, Zhang LM, Rasmussen SK, et al Stability and genetic control of morphological, biomass and biofuel traits under temperate maritime and continental conditions in sweet sorghum (Sorghum bicolour). Theor Appl Genet. 2015;128(9):1685–701. 10.1007/s00122-015-2538-5 25982132

[21] Houston K, Burton RA, Sznajder B, Rafalski AJ, Dhugga KS, Mather DE, et al A Genome-Wide Association Study for Culm Cellulose Content in Barley Reveals Candidate Genes Co-Expressed with Members of the CELLULOSE SYNTHASE A Gene Family. PLoS One. 2015;10(7). 10.1371/journal.pone.0130890 PMC4496100 26154104

[22] Moller I, Sorensen I, Bernal AJ, Blaukopf C, Lee K, Obro J, et al High-throughput mapping of cell-wall polymers within and between plants using novel microarrays. Plant J. 2007;50(6):1118–28. 10.1111/j.1365-313X.2007.03114.x 17565618

[23] Tyler L, Fangel JU, Fagerstrom AD, Steinwand MA, Raab TK, Willats WGT, et al Selection and phenotypic characterization of a core collection of Brachypodium distachyon inbred lines. Bmc Plant Biol. 2014;14. 10.1186/1471-2229-14-25 PMC3925370 24423101

[24] Alonso-Simon A, Kristensen JB, Obro J, Felby C, Willats WGT, Jorgensen H. High-Throughput Microarray Profiling of Cell Wall Polymers During Hydrothermal Pre-Treatment of Wheat Straw. Biotechnol Bioeng. 2010;105(3):509–14. 10.1002/bit.22546 19777595

[25] Scholthof KBG, Adkins S, Czosnek H, Palukaitis P, Jacquot E, Hohn T, et al Top 10 plant viruses in molecular plant pathology. Mol Plant Pathol. 2011;12(9):938–54. 10.1111/j.1364-3703.2011.00752.x 22017770 PMC6640423

[26] Technow F. R Package for moving grid adjustment in plant breeding field trials. 2014 [October 10, 2014]. http://cran.r-project.org/web/packages/mvngGrAd/index.html.

[27] Lado B, Matus I, Rodriguez A, Inostroza L, Poland J, Belzile F, et al Increased Genomic Prediction Accuracy in Wheat Breeding Through Spatial Adjustment of Field Trial Data. G3-Genes Genom Genet. 2013;3(12):2105–14. 10.1534/g3.113.007807 PMC3852373 24082033

[28] Bates D, Maechler M, Bolker B, Walker S. lme4: Linear mixed-effects models using Eigen and S4 2014 [cited 2014]. http://CRAN.R-project.org/package=lme4.

[29] Akar T, Francia E, Tondelli A, Rizza F, Stanca AM, Pecchioni N. Marker-assisted characterization of frost tolerance in barley (Hordeum vulgare L.). Plant Breeding. 2009;128(4):381–6.

[30] Leplat F, Jensen J, Madsen P. Genomic Prediction of Manganese Efficiency in Winter Barley. Plant Genome-Us. 2016;9(2). 10.3835/plantgenome2015.09.0085 27898822

[31] Digel B, Tavakol E, Verderio G, Tondelli A, Xu X, Cattivelli L, et al Photoperiod-H1 (Ppd-H1) Controls Leaf Size. Plant Physiol. 2016;172(1):405–15. 10.1104/pp.16.00977 27457126 PMC5074620

[32] Schwender H. Imputing Missing Genotypes with Weighted K Nearest Neighbors. J Toxicol Env Heal A. 2012;75(8–10):438–46. 10.1080/15287394.2012.674910 22686303

[33] Munoz-Amatriain M, Cuesta-Marcos A, Endelman JB, Comadran J, Bonman JM, Bockelman HE, et al The USDA Barley Core Collection: Genetic Diversity, Population Structure, and Potential for Genome-Wide Association Studies. PLoS One. 2014;9(4). 10.1371/journal.pone.0094688 PMC3986206 24732668

[34] Pritchard JK, Stephens M, Donnelly P. Inference of population structure using multilocus genotype data. Genetics. 2000;155(2):945–59. 10835412 10.1093/genetics/155.2.945 PMC1461096

[35] Evanno G, Regnaut S, Goudet J. Detecting the number of clusters of individuals using the software STRUCTURE: a simulation study. Mol Ecol. 2005;14(8):2611–20. 10.1111/j.1365-294X.2005.02553.x 15969739

[36] Earl DA, Vonholdt BM. STRUCTURE HARVESTER: a website and program for visualizing STRUCTURE output and implementing the Evanno method. Conserv Genet Resour. 2012;4(2):359–61.

[37] Jakobsson M, Rosenberg NA. CLUMPP: a cluster matching and permutation program for dealing with label switching and multimodality in analysis of population structure. Bioinformatics. 2007;23(14):1801–6. 10.1093/bioinformatics/btm233 17485429

[38] Bradbury PJ, Zhang Z, Kroon DE, Casstevens TM, Ramdoss Y, Buckler ES. TASSEL: software for association mapping of complex traits in diverse samples. Bioinformatics. 2007;23(19):2633–5. 10.1093/bioinformatics/btm308 17586829

[39] Nielsen NH, Backes G, Stougaard J, Andersen SU, Jahoor A. Genetic Diversity and Population Structure Analysis of European Hexaploid Bread Wheat (Triticum aestivum L.) Varieties. PLoS One. 2014;9(4). 10.1371/journal.pone.0094000 PMC3981729 24718292

[40] Lipka AE, Tian F, Wang QS, Peiffer J, Li M, Bradbury PJ, et al GAPIT: genome association and prediction integrated tool. Bioinformatics. 2012;28(18):2397–9. 10.1093/bioinformatics/bts444 22796960

[41] Houston K, Russell J, Schreiber M, Halpin C, Oakey H, Washington JM, et al A genome wide association scan for (1,3;1,4)-beta-glucan content in the grain of contemporary 2-row Spring and Winter barleys. BMC Genomics. 2014;15. 10.1186/1471-2164-15-907 PMC4213503 25326272

[42] Marcotuli I, Houston K, Waugh R, Fincher GB, Burton RA, Blanco A, et al Genome Wide Association Mapping for Arabinoxylan Content in a Collection of Tetraploid Wheats. PLoS One. 2015;10(7). 10.1371/journal.pone.0132787 PMC4503733 26176552

[43] Stein N, Perovic D, Kumlehn J, Pellio B, Stracke S, Streng S, et al The eukaryotic translation initiation factor 4E confers multiallelic recessive Bymovirus resistance in Hordeum vulgare (L.). Plant J. 2005;42(6):912–22. 10.1111/j.1365-313X.2005.02424.x 15941403

[44] Sedlacek T, Marik P, Chrpova J. Development of CAPS Marker for Identification of rym4 and rym5 Alleles Conferring Resistance to the Barley Yellow Mosaic Virus Complex in Barley. Czech J Genet Plant Breed. 2010;46(4):159–63.

[45] Perovic D, Kramer I, Habekuss A, Perner K, Pickering R, Proeseler G, et al Genetic analyses of BaMMV/BaYMV resistance in barley accession HOR4224 result in the identification of an allele of the translation initiation factor 4e (Hv-eIF4E) exclusively effective against Barley mild mosaic virus (BaMMV). Theor Appl Genet. 2014;127(5):1061–71. 10.1007/s00122-014-2279-x 24522725

[46] Yang P, Habekuss A, Ordon F, Stein N. Analysis of bymovirus resistance genes on proximal barley chromosome 4HL provides the basis for precision breeding for BaMMV/BaYMV resistance. Theor Appl Genet. 2014;127(7):1625–34. 10.1007/s00122-014-2324-9 24849455

[47] Peltonen-Sainio P, Jauhiainen L, Laurila IP. Cereal yield trends in northern European conditions: Changes in yield potential and its realisation. Field Crop Res. 2009;110(1):85–90.

[48] Dwivedi SL, Crouch JH, Mackill DJ, Xu Y, Blair MW, Ragot M, et al The molecularization of public sector crop breeding: Progress, problems, and prospects In: Sparks DL, editor. Advances in Agronomy, Vol 95 Advances in Agronomy. 95. San Diego: Elsevier Academic Press Inc; 2007 p. 163–318.

[49] Kai H, Takata K, Tsukazaki M, Furusho M, Baba T. Molecular mapping of Rym17, a dominant and rym18 a recessive barley yellow mosaic virus (BaYMV) resistance genes derived from Hordeum vulgare L. Theor Appl Genet. 2012;124(3):577–83. 10.1007/s00122-011-1730-5 22038435

[50] Kumlehn J, Stein N, SpringerLink (Online service). Biotechnological Approaches to Barley Improvement. http://proxy.library.cornell.edu/login?url=http://link.springer.com/openurl?genre=book&isbn=978-3-662-44405-4.

[51] Kuczynska A, Surma M, Adamski T, Mikolajczak K, Krystkowiak K, Ogrodowicz P. Effects of the semi-dwarfing sdw1/denso gene in barley. J Appl Genet. 2013;54(4):381–90. 10.1007/s13353-013-0165-x 23975516 PMC3825292

[52] Malosetti M, van Eeuwijk FA, Boer MP, Casas AM, Elia M, Moralejo M, et al Gene and QTL detection in a three-way barley cross under selection by a mixed model with kinship information using SNPs. Theor Appl Genet. 2011;122(8):1605–16. 10.1007/s00122-011-1558-z 21373796 PMC3082036

[53] Sang J, Zhang Z, Wu G. Gene: "Os01g0883800" in RiceWiki 2015. http://ricewiki.big.ac.cn/index.php/Os01g0883800.

[54] Tyrka M, Perovic D, Wardynska A, Ordon F. A new diagnostic SSR marker for selection of the Rym4/Rym5 locus in barley breeding. J Appl Genetics. 2008;49(2):127–34. 18436986 10.1007/BF03195605

[55] Houston K, McKim SM, Comadran J, Bonar N, Druka I, Uzrek N, et al Variation in the interaction between alleles of HvAPETALA2 and microRNA172 determines the density of grains on the barley inflorescence. P Natl Acad Sci USA. 2013;110(41):16675–80. 10.1073/pnas.1311681110 PMC3799380 24065816

[56] Lopes MS, Dreisigacker S, Pena RJ, Sukumaran S, Reynolds MP. Genetic characterization of the wheat association mapping initiative (WAMI) panel for dissection of complex traits in spring wheat. Theor Appl Genet. 2015;128(3):453–64. 10.1007/s00122-014-2444-2 25540818

[57] Mikkelsen D, Flanagan BM, Wilson SM, Bacic A, Gidley MJ. Interactions of Arabinoxylan and (1,3)(1,4)-beta-Glucan with Cellulose Networks. Biomacromolecules. 2015;16(4):1232–9. 10.1021/acs.biomac.5b00009 25756836

[58] Mikkelsen MD, Harholt J, Ulvskov P, Johansen IE, Fangel JU, Doblin MS, et al Evidence for land plant cell wall biosynthetic mechanisms in charophyte green algae. Ann Bot-London. 2014;114(6):1217–36. 10.1093/aob/mcu171 PMC4195564 25204387

[59] Pedersen HL, Fangel JU, McCleary B, Ruzanski C, Rydahl MG, Ralet M-C, et al Versatile High Resolution Oligosaccharide Microarrays for Plant Glycobiology and Cell Wall Research. Journal of Biological Chemistry. 2012;287(47). 10.1074/jbc.M112.396598 PMC3501085 22988248

[60] Bailoni L, Bonsembiante M, Schiavon S, Pagnin G, Tagliapietra F. Estimation of the content of pectins in feeds: Fractional extraction and quantitative determination. Vet Res Commun. 2003;27:249–51. 14535402 10.1023/b:verc.0000014152.80334.86

[61] Seifert GJ, Roberts K. The biology of arabinogalactan proteins. Annu Rev Plant Biol. 2007;58:137–61. 10.1146/annurev.arplant.58.032806.103801 17201686

[62] Tan L, Showalter AM, Egelund J, Hernandez-Sanchez A, Doblin MS, Bacic A. Arabinogalactan-proteins and the research challenges for these enigmatic plant cell surface proteoglycans. Frontiers in Plant Science. 2012;3. 10.3389/fpls.2012.00140 PMC3384089 22754559

[63] Marzec M, Szarejko I, Melzer M. Arabinogalactan proteins are involved in root hair development in barley. J Exp Bot. 2015;66(5):1245–57. 10.1093/jxb/eru475 25465033 PMC4339589

[64] Uma B, Rani TS, Podile AR. Warriors at the gate that never sleep: Non-host resistance in plants. J Plant Physiol. 2011;168(18):2141–52. 10.1016/j.jplph.2011.09.005 22001579

[65] Zhang ZY, Xin WW, Wang SF, Zhang X, Dai HF, Sun RR, et al Xylem sap in cotton contains proteins that contribute to environmental stress response and cell wall development. Funct Integr Genomics. 2015;15(1):17–26. 10.1007/s10142-014-0395-y 25163431

[66] Alexandersson E, Jacobson D, Vivier MA, Weckwerth W, Andreasson E. Field-omics-understanding large-scale molecular data from field crops. Frontiers in Plant Science. 2014;5. 10.3389/fpls.2014.00286 PMC4064663 24999347

[67] Brown TB, Cheng RY, Sirault XRR, Rungrat T, Murray KD, Trtilek M, et al TraitCapture: genomic and environment modelling of plant phenomic data. Curr Opin Plant Biol. 2014;18:73–9. 10.1016/j.pbi.2014.02.002 24646691

[68] Granier C, Vile D. Phenotyping and beyond: modelling the relationships between traits. Curr Opin Plant Biol. 2014;18:96–102. 10.1016/j.pbi.2014.02.009 24637194

[69] Leplat F, Pedas PR, Rasmussen SK, Husted S. Identification of manganese efficiency candidate genes in winter barley (Hordeum vulgare) using genome wide association mapping. BMC Genomics. 2016;17:15. 27716061 10.1186/s12864-016-3129-9 PMC5050567

