# Supplementary figures and images for: Genome-wide association mapping in winter barley for grain yield and culm cell wall polymer content using the high-throughput CoMPP technique
Source: PLoS One. 2017 Mar 16;12(3):e0173313. doi: 10.1371/journal.pone.0173313 (PMC5354286; doi:10.1371/journal.pone.0173313)

**GYLD 2009**

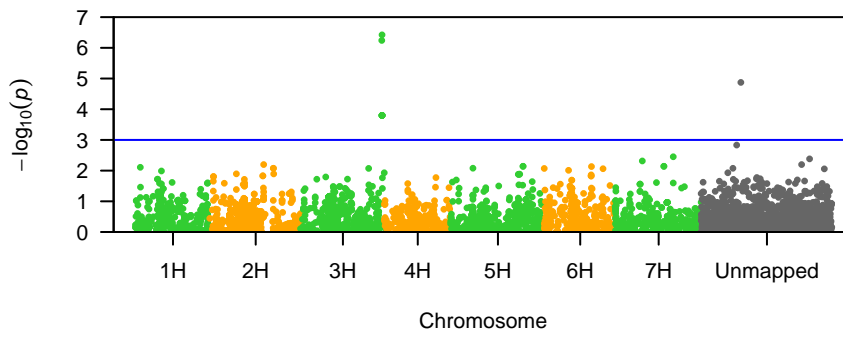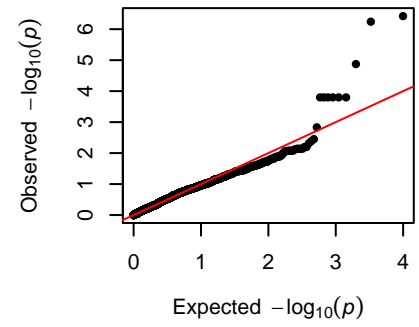

**GYLD 2010**

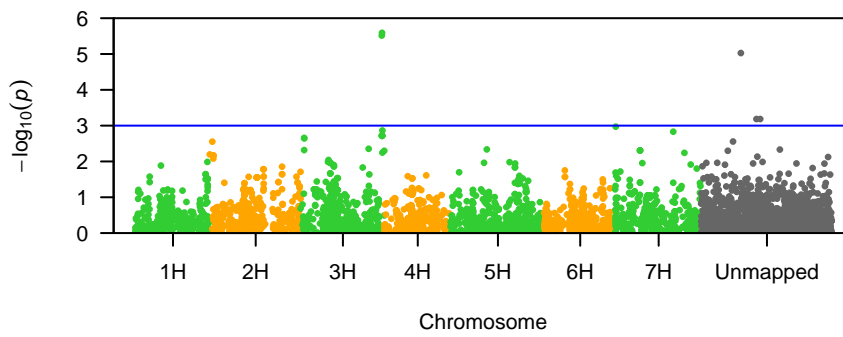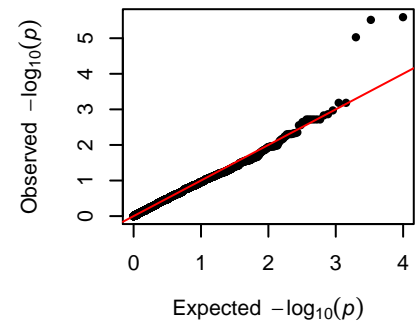

**GYLD 09+10**

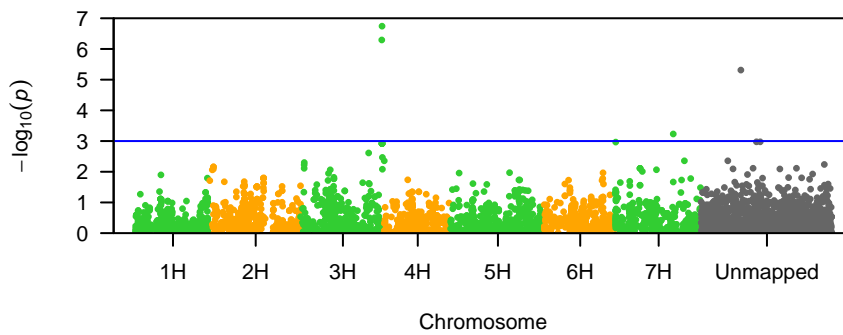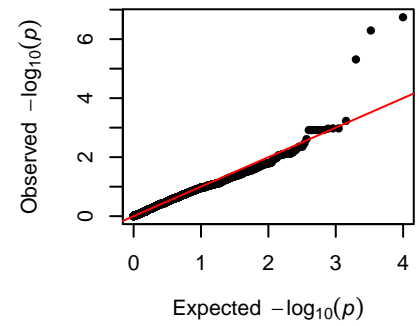

**LM6 2009**

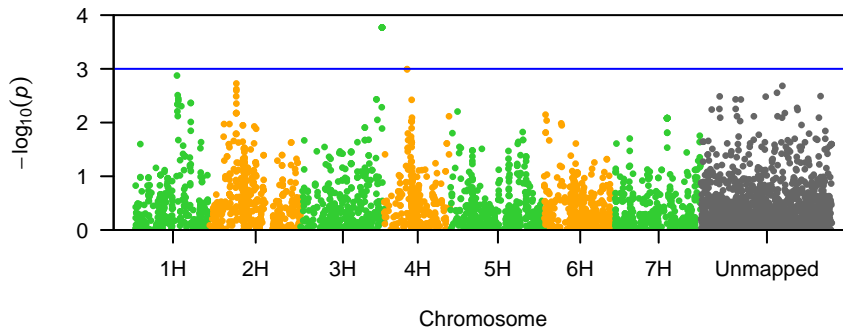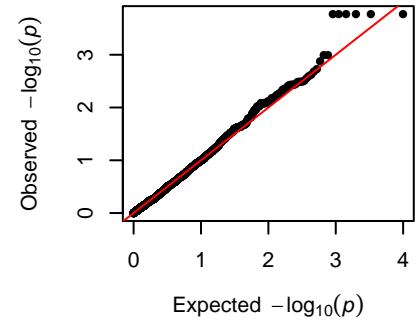

**LM6 2010**

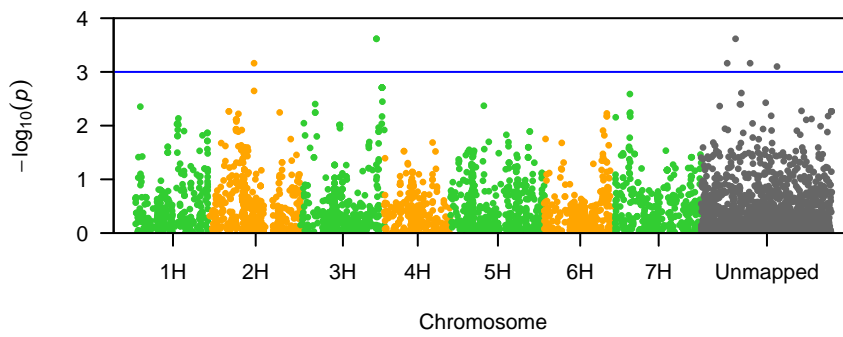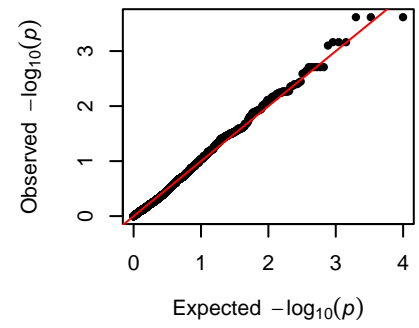

**LM6 09+10**

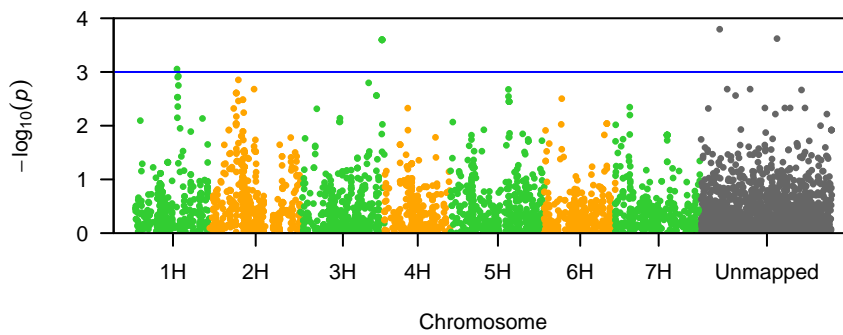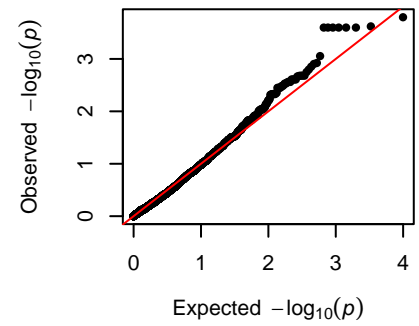

**JIM13 2009**

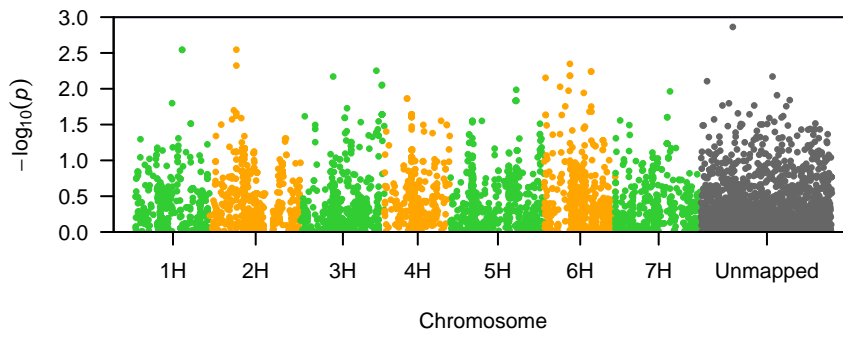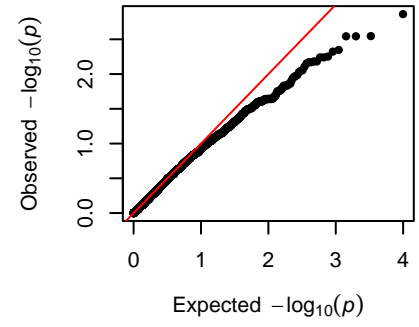

**JIM13 2010**

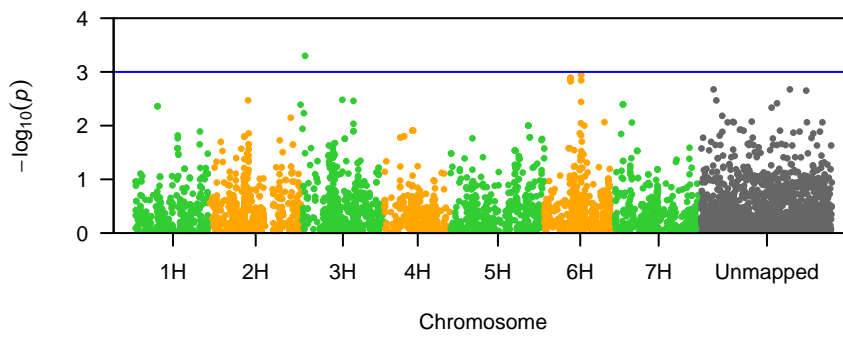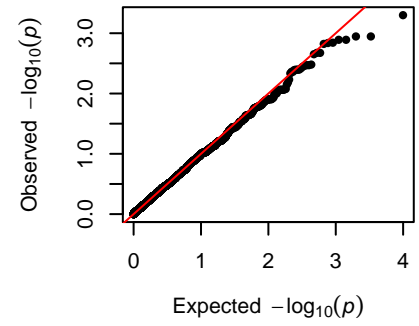

**JIM13 09+10**

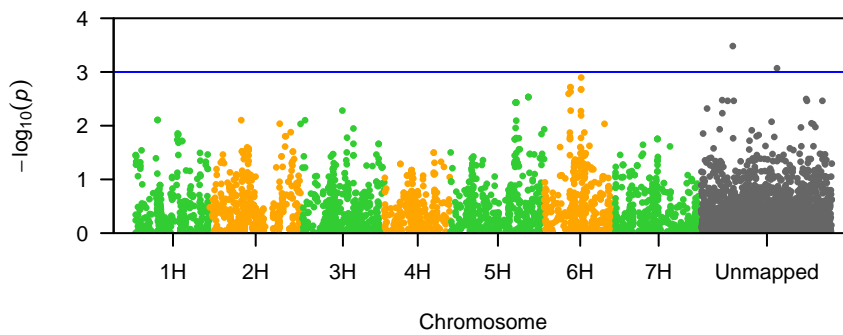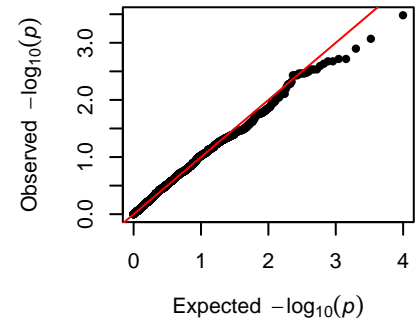

Supplement: S1 Fig — (PDF) [file pone.0173313.s001.pdf]
